# *Trans*-regulatory variant network contributes to missing heritability

**DOI:** 10.1016/j.xgen.2023.100470

**Published:** 2024-01-10

**Authors:** Vanessa Pereira, Elena Kuzmin

**Affiliations:** 1 Department of Biology, Concordia University, Montreal, Canada; 2 Centre for Applied Synthetic Biology, Centre for Structural and Functional Genomics, Concordia University, Montreal, Canada; 3 Department of Human Genetics, Rosalind & Morris Goodman Cancer Institute, McGill University, Montreal, Canada

## Abstract

In a recent *Cell Genomics* article, Tsouris et al.^1^ analyze the transcriptomes of a large diallel panel of hybrids from *Saccharomyces cerevisiae* natural isolates to study *cis*- and *trans*-regulatory changes underlying gene expression variation. Vanessa Pereira and Elena Kuzmin discuss the authors’ findings and the wider context in missing heritability research in this preview.

## Teaser

In a recent *Cell Genomics* article, Tsouris et al. analyze the transcriptomes of a large diallel panel of hybrids from *Saccharomyces cerevisiae* natural isolates to study *cis*- and *trans*-regulatory changes underlying gene expression variation. Vanessa Pereira and Elena Kuzmin discuss the authors’ findings and the wider context in missing heritability research in this preview.

## Main text

Gene expression regulation has important consequences on organismal phenotypes.^2^ Variation of gene expression among individuals of a natural population is useful for understanding the genotype-to-phenotype relationship. Regulatory variants involving *cis*-acting factors (mutations in promoter regions) or *trans*-acting factors (mutations in transcription factors) and their interplay underlie heritable gene expression variation in a population. Large-scale genomic and transcriptomic analyses are used to measure statistical associations between genetic variants and gene expression levels, thereby identifying *cis*- and *trans*-regulatory elements. Detection of expression quantitative trait loci (eQTL), including those using human genome-wide association studies, requires a large sample size to achieve sufficient statistical power.^3^ This limits the discovery of *trans*- compared to *cis*-regulatory effects since the former requires many more possible positions to test. However, failing to account for these *trans* factors and their interactions with *cis* factors can contribute to the observed missing heritability.^4^^,^^5^

**Figure 1:**
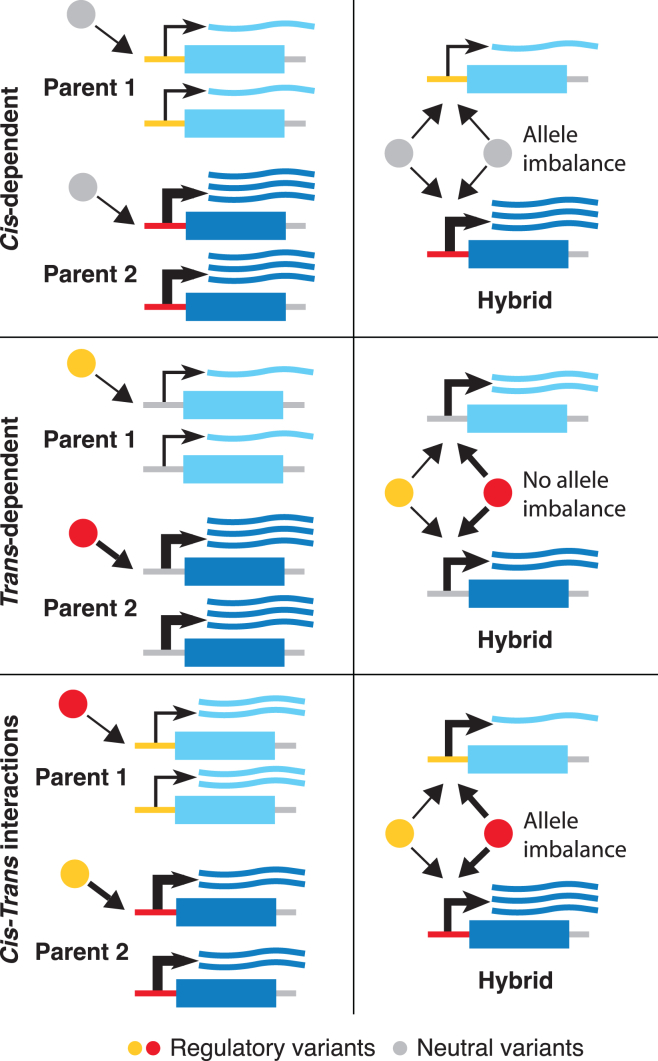
*Cis*- and *trans*-regulatory effects across a population Illustration of *cis*-, *trans*-, and *cis*-*trans*-regulatory variation and the resulting allele-specific expression patterns across parent-hybrid trios. Top: single-nucleotide polymorphisms (SNPs) in the (top) local regulatory elements (*cis*-regulatory change) manifest as different expression levels in both parents and hybrids. Middle: SNPs in the distant regulatory genes (*trans*-regulatory change) manifest as different parental expression levels but have no difference in allele expression levels in the hybrid. Bottom: SNPs in both local and distant regulatory elements show a complex pattern of expression due to *cis*-*trans* interactions. Compensatory effects are shown as an example.

Allele-specific expression (ASE) analysis using purebred parental lines and their F1 hybrids to quantify the relative expression of two alleles in a diploid individual offers an alternative approach to detect *cis*- and *trans*-regulatory variants. Compared to eQTL studies, ASE-based studies focus on gene-level regulatory changes and thus are statistically well powered. By focusing on parent-hybrid trios, ASE-based studies provide an advantage since they use parent-offspring regression to infer heritability and capture both additive and non-additive variation (Figure 1). A previous study in yeast used a large biparental segregant panel and found evidence to suggest a significant contribution of non-additive effect on gene expression.^3^ ASE-based studies have been extensively conducted in other organisms; however, they were often limited to one or a few parent-hybrid trios.

To identify *trans*-eQLTs in a large-scale ASE-based study, Tsouris et al.^1^ conducted transcriptomic analysis of a large diallel panel of 323 F1 hybrids from 26 *S*. *cerevisiae* isolates. They were obtained from the 1,011-yeast collection of natural isolates capturing the genomic diversity of the species.^6^ RNA sequencing was carried out on the heterozygous hybrids and homozygous parental lines obtaining overall and allele-specific expression levels for 6,186 genes. The authors analyzed core genes that are present in all these parental lines and accessory genes, most of which originate from *S. paradoxus* introgression.

The diallel experimental design enabled authors to calculate the narrow-sense heritability (*h*^*2*^), which refers to the phenotypic variance due to additive effects of genes, and broad-sense heritability (*H*^*2*^), which refers to total genetic contribution to the phenotypic variance of a population, for each expression trait. This design thus allowed them to measure the additive contribution from the parental lines or the non-additive contribution from the parental combination in the hybrid. Non-additive variance was, on average, responsible for 36% of phenotypic variance controlling the expression of one-third of the genes, whereas additive variance controlled one-tenth of the genes and the variation of expression of one-fifth of the genes was stochastic. Non-additive genes were enriched for biological processes such as translation, ribosome biogenesis, and sulfur amino acid biosynthesis, while additive genes were enriched for protein transport to the vacuole. No enrichment was seen for genes with stochastic variation, indicating functional preference for a specific type of regulation. Genes annotated to terms that were enriched for non-additive variance showed higher correlated expression profiles across hybrids than highly additive genes, suggesting that non-additive genes are co-regulated across hybrids. These findings also suggest that the non-additive variance component is more functionally significant than additive.

The authors then sought to determine whether regulatory variation was in *cis* or in *trans* by comparing allelic expression in the hybrid to the expression levels in two parental lines (hybrid-parent trio). A *cis*-regulatory change was identified when the parental lines that differed in expression resulted in an allele-specific expression in the hybrid. On the other hand, a *trans*-regulatory change showed no allele-specific expression because the *trans*-acting factor influences both alleles equally in the hybrid. Examining ∼1.2 million sites across 285,777 gene-trio combinations revealed that one-fourth of cases showed allelic expression difference between the hybrid and the parents, with84% due exclusively to *trans* effects, 4% due exclusively to *cis* effects, and 12% due to both *cis* and *trans* effects.

Four distinct regulatory patterns were identified: the “attenuating” group in which *trans* factors decrease *cis* effects in the hybrid compared to parental expression levels, the “reinforcing” group in which *trans* factors increase *cis* effects, the most common “compensatory” group *trans* factors cancel out the *cis* effects, and the “reverse” group with extreme *cis*-*trans* interactions. Overall, regulatory variation in *trans* was more common than in *cis*, which was consistent with previous studies.^3^^,^^7^^,^^8^ Most *cis* effects are modulated by *trans* effects, which often act in the opposite direction, suggesting gene-expression-buffering mechanisms. Compensatory patterns were enriched for genes annotated to translation, indicating a mechanism for the buffering of this process. *Trans*-regulated genes were also more highly expressed, less dispersed, and more highly connected on the co-expression network than *cis*-regulated genes, highlighting their functional significances. Compensatory and *trans*-regulated genes also showed more non-additive variance compared to *cis*-controlled genes.

Approximately one-third of *S. cerevisiae* accessory genes arise from *S. paradoxus* introgression.^6^ Since the diallel panel included two Alpechin isolates with introgressed genes, the authors compared regulatory variation within (*S. cer* vs. *S. cer*) and between species (*S. cer* vs. *S. par*). Between-species allele pairs show more *cis*-regulatory variation than within-species allele pairs largely due to many *cis*-regulatory changes between species. Most genes had conserved regulatory patterns, and the majority of those showed *trans*-regulation for the introgressed allele and *S. cer* allele. The authors highlighted that *trans* factors controlled the *S. cer* allele of the *cis*-regulated introgressed alleles. Introgressed genes with conserved regulatory patterns showed a higher connectivity in the gene expression network and lower additive variance. Thus, heritable variation of introgressed genes may be influenced not only by interspecies differences but also by their global connectivity.

In conclusion, the study by Tsouris et al.^1^ represents a significant advance in our understanding of the regulatory landscape of trait heritability. The identification of *trans* factors as the main driver underlying non-additive variance highlights the importance of considering *trans*-regulatory changes in future genetic association studies. Their integration can help uncover additional genetic factors contributing to the heritability of gene expression variation and thus improve our understanding of phenotypic variation. Although further research is needed to elucidate the precise mechanisms, the implication of genes with high non-additive variance being highly connected on the global gene expression network and being *trans*-regulated suggests that non-additive variance and its buffering effect contribute to missing heritability. Furthermore, together with previous research,^9^ the Tsouris et al. study highlights two major routes for *trans*-regulation for gene expression variance through either *cis*-*trans* interactions or coordinated expression change. As sequencing technologies continue to advance and become more accessible, we can anticipate that analysis of additional *S. cer* isolates and growth conditions, as well as more nuanced integration of regulatory networks with genetic interaction networks,^10^ will greatly enhance our understanding of the biology of inheritance.

## References

[1] Tsouris A., Brach G., Schacherer J., Hou J. (2023). Non-additive genetic components contribute significantly to population-wide gene expression variation. Cell Genom..

[2] Albert F.W., Kruglyak L. (2015). The role of regulatory variation in complex traits and disease. Nat. Rev. Genet..

[3] Albert F.W., Bloom J.S., Siegel J., Day L., Kruglyak L. (2018). Genetics of trans-regulatory variation in gene expression. elife.

[4] Manolio T.A., Collins F.S., Cox N.J., Goldstein D.B., Hindorff L.A., Hunter D.J., McCarthy M.I., Ramos E.M., Cardon L.R., Chakravarti A. (2009). Finding the missing heritability of complex diseases. Nature.

[5] Zuk O., Hechter E., Sunyaev S.R., Lander E.S. (2012). The mystery of missing heritability: Genetic interactions create phantom heritability. Proc. Natl. Acad. Sci. USA.

[6] Peter J., De Chiara M., Friedrich A., Yue J.X., Pflieger D., Bergström A., Sigwalt A., Barre B., Freel K., Llored A. (2018). Genome evolution across 1,011 Saccharomyces cerevisiae isolates. Nature.

[7] Wittkopp P.J., Haerum B.K., Clark A.G. (2004). Evolutionary changes in cis and trans gene regulation. Nature.

[8] Schaefke B., Emerson J.J., Wang T.Y., Lu M.Y.J., Hsieh L.C., Li W.H. (2013). Inheritance of gene expression level and selective constraints on trans- and cis-regulatory changes in yeast. Mol. Biol. Evol..

[9] Landry C.R., Wittkopp P.J., Taubes C.H., Ranz J.M., Clark A.G., Hartl D.L. (2005). Compensatory cis-trans evolution and the dysregulation of gene expression in interspecific hybrids of Drosophila. Genetics.

[10] Costanzo M., Kuzmin E., van Leeuwen J., Mair B., Moffat J., Boone C., Andrews B. (2019). Global Genetic Networks and the Genotype-to-Phenotype Relationship. Cell.

